# Case report: Long-term follow-up of patients who received a FimCH vaccine for prevention of recurrent urinary tract infections caused by antibiotic resistant Enterobacteriaceae: a case report series

**DOI:** 10.3389/fimmu.2024.1359738

**Published:** 2024-03-13

**Authors:** Elise Perer, Helen Stacey, Terri Eichorn, Heidi Hughey, Julie Lawrence, Eric Cunningham, Mark O’Neil Johnson, Kevin Bacon, Andrew Kau, Scott J. Hultgren, Thomas M. Hooton, Jennifer L. Harris

**Affiliations:** ^1^Family Medicine Associates at Northridge, Northridge, CA, United States; ^2^Diablo Clinical Research, Walnut Creek, CA, United States; ^3^Sequoia Vaccines, Inc., St. Louis, MO, United States; ^4^Department of Molecular Microbiology and Center for Women’s Infectious Disease Research, Washington University School of Medicine, St. Louis, MO, United States; ^5^Department of Medicine, School of Medicine, University of Miami, Miami, FL, United States

**Keywords:** urinary tract infection, vaccine, FimH, expanded access, carbapenem-resistant Enterobacteriaceae

## Abstract

Urinary tract infections (UTI) caused by carbapenem-resistant Enterobacteriaceae (CRE) are considered one of the most urgent health threats to humans according to the Centers for Disease Control (CDC), and the World Health Organization (WHO). A FimCH Vaccine expanded access study is being conducted in patients with a history of antibiotic resistant UTIs who are considered to be at risk for development of CRE UTI. This case series describes the clinical, safety and immunogenicity findings for four participants who received a FimCH four-vaccine series. Participants were followed for 12 months after administration of the fourth vaccine for safety, general health status and UTI occurrence. The study was later amended to allow additional follow-up of up to five years post vaccine administration to assess long-term health status, UTI occurrences and to obtain blood samples for anti-FimH antibody testing. In our population of 4 study participants, the number of symptomatic UTI occurrences caused by gram-negative bacteria in the 12-month period following peak anti-FimH antibody response were approximately 75% lower than the 12-month period preceding study enrollment. These results are consistent with the 30-patient cohort of a Phase 1 study with the same FimCH Vaccine. UTI occurrences increased during the long-term follow-up period for all 4 participants but did not reach the rate observed pre-vaccination. No new safety concerns related to the FimCH Vaccine were identified during long-term follow-up. This case series has clinical importance and public health relevance since it examines and reports on UTI frequency and recurrence following vaccination with the FimCH Vaccine in a high-risk population of patients with recurrent UTI. Additionally, participants described improved well-being following vaccination which was maintained in the long-term follow-up period.

## Introduction

Urinary tract infections (UTIs) are common bacterial infections; estimated to occur in greater than half of women within their lifetime with up to 30% of women experiencing a recurrent UTI within 3-4 months of the initial event (1, 2). While UTIs are less common in men, UTI frequency increases with age in both men and women (3). UTIs are the most common infection that leads to antibiotic use following a visit to a physician (4). The majority of UTIs are caused by gram-negative bacteria with *Escherichia coli (E. coli)* and *Klebsiella* spp. being the predominant bacteria identified from clean-catch midstream urine collections (5). The rise of resistant strains of bacteria associated with UTIs, including extended spectrum beta-lactamase (ESBL)-producing strains, complicates treatment, and often necessitates hospitalization for intravenous antibiotic treatment (6). Moreover, evidence suggests that UTIs are the origin of more than 25% of sepsis cases (7).

The economic burden of UTIs is significant with estimated annual treatment costs of 3.5 billion dollars in the United States (8). Additionally, patients with recurrent UTIs commonly report anxiety, depression and frequent missed workdays resulting in substantial psychosocial and societal burden (9).

## FimCH vaccine

The antigen of the FimCH Vaccine being developed by Sequoia Vaccines, Inc., is a complex of two proteins, FimC and FimH. FimH is a mannose-binding adhesin protein. FimC is a chaperone protein that stabilizes FimH. The FimCH complex is formulated with a synthetic adjuvant, Phosphorylated HexaAcyl Disaccharide (PHAD).

The FimCH Vaccine development program is designed to evaluate the ability of the FimCH Vaccine to prevent recurrent urinary tract infections caused by *E. coli* and other bacteria in the Enterobacteriaceae family responsible for UTIs by preventing type 1 pilus-mediated attachment to bladder epithelial cells. While *E. coli* is the most common pathogen associated with UTIs, many other species in the Enterobacteriaceae family encode type 1 pili (10). FimCH has undergone pre-clinical and clinical evaluations including a Phase 1 trial evaluating safety and immunogenicity which included healthy volunteers and 30 women with a history of recurrent UTI (11).

## Study description

The Sequoia FimCH Expanded Access Study (EAS) is a clinical, open-label study conducted under an IND program. The EAS is designed to evaluate the safety, tolerability, and immunogenicity of the FimCH Vaccine when administered as an intramuscular (IM) injection in adult participants at risk for developing multidrug, antibiotic resistant Enterobacteriaceae UTI requiring carbapenem antibiotic treatment.

Patients with a history of recurrent UTI are screened to assess their eligibility within a 30-day Enrollment/Screening period prior to administration of the FimCH Vaccine which includes a physical exam, a comprehensive metabolic panel (CMP), complete blood count (CBC) and review of medical history. Patients without acute or chronic medical conditions, or clinically significant laboratory values, which in the opinion of the investigator would make vaccination unsafe, are eligible for participation.

The FimCH Vaccine consists of a series of four injections of 107 µg FimCH (antigen) plus 43 µg PHAD (adjuvant) at Day 1, Day 30, Day 90, and Day 180. Participants remain at the clinical site for a post-dose observation period of approximately 2 hours after the first dose and for a minimum of 30 minutes for subsequent doses for evaluation of acute injection site reaction adverse events.

Participants are followed for approximately 12 months following administration of the fourth vaccine of the series. The study was later amended to evaluate additional long-term follow-up of participants up to 5 years. As part of the long-term follow-up visit, participant interviews are conducted to gain patient perspective regarding general well-being immediately following vaccination with the FimCH Vaccine and during the long-term follow-up period up to five years post vaccination.

The EAS Study was initially approved by Schulman/Advarra IRB on 14 November 2017 with subsequent approvals for each amendment including the amendment incorporating the optional long-term follow-up on 10 May 2023.

The EAS has enrolled a total of 5 patients as of December 2023 with four completing the study per protocol requirements. Here we report 12-month follow-up after completion of the four-vaccine series and additional long-term follow-up for general medical health status, UTI history, and blood collection for anti-FimH antibody testing for the four completed participants.

## Participant case narratives

### Participant 1: 10-08-001

At the time of screening for the Sequoia FimCH EAS participant 10-08-001, a 73-year-old female presented with a history of 5 symptomatic gram-negative culture-verified UTIs in the previous 12-monthes. She had experienced multiple UTI antibiotic treatment failures, and her physician considered her to be at risk of requiring intravenous carbapenem for subsequent UTIs. Her medical history did not include exclusionary comorbidities. No infections requiring antibiotics were reported in the 12 months prior to study enrollment or during the post-vaccination period other than UTIs.

**Figure 1:**
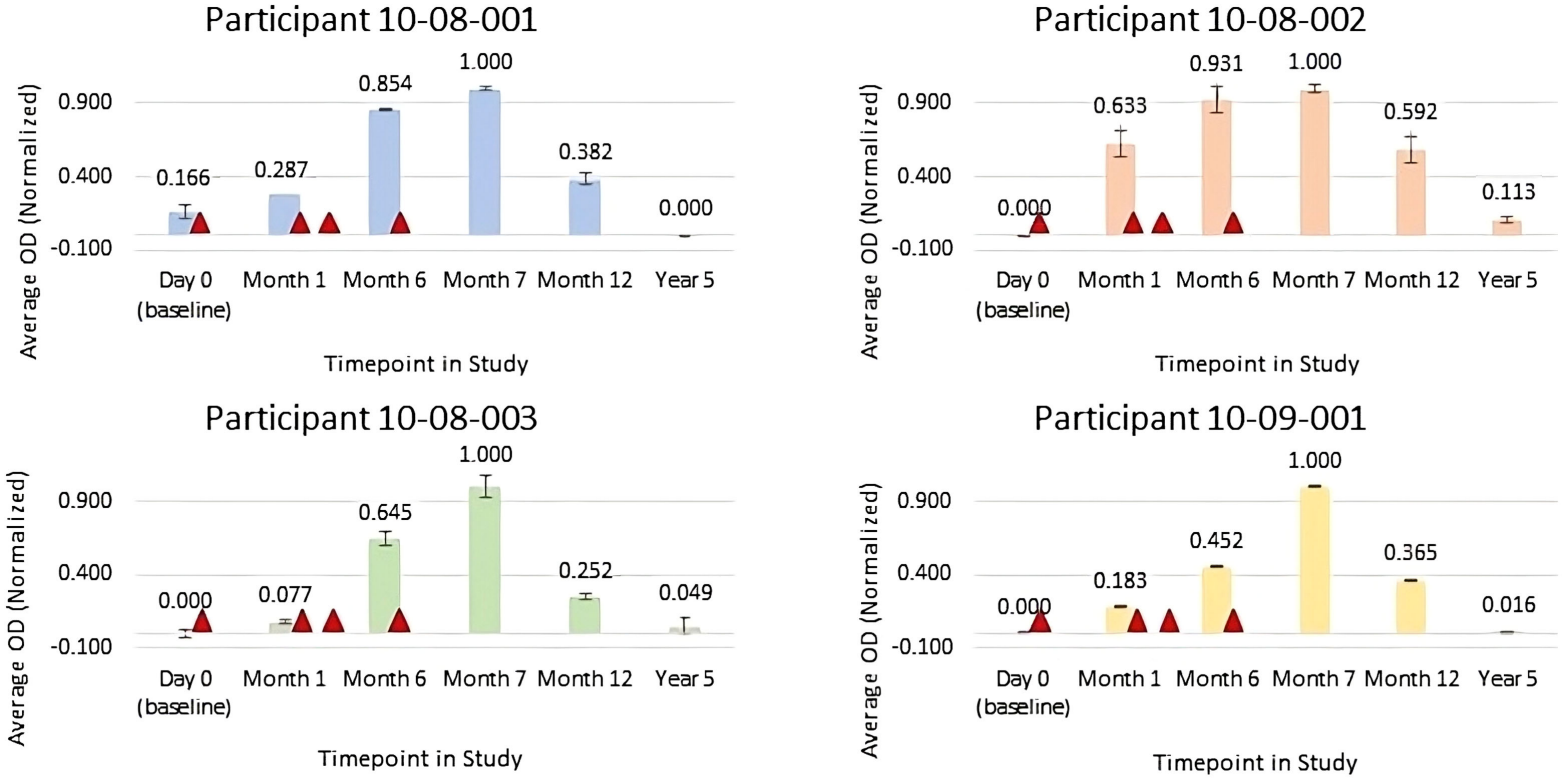
Normalized average anti-FimH IgG Optical Density (OD) for serum samples collected pre-vaccination (Day 0), at month 1 (prior to FimCH vaccine dose #2), month 6 (prior to FimCH vaccine dose #4, month 7 (30 days post FimCH vaccine dose #4), and year 5 (approximately 4 years post FimCH vaccine dose #4). Vaccine dosing is indicated by ▲ Day 1, Day 30, Day 90 and Day 180 on study for each participant.

Participant 10-08-001 was consented on 6 March 2018. A series of four FimCH vaccines were administered on 6 March 2018, 10 April 2018, 11 June 2018, and 28 August 2018 per protocol. During the 12-month period from study month 7 to Month 19, participant 10-08-001 was diagnosed with 3 symptomatic gram-negative UTIs confirmed by culture for multi-drug resistant *E. coli* and *Klebsiella*. The decrease in symptomatic culture-verified UTI occurrences post vaccine correlates with observed peak anti-FimH antibody response (**Figure 1**). She did not report any vaccine related adverse events, unexpected adverse events or serious adverse events during study participation. She completed the EAS on 19 September 2019.

Participant 10-08-001 consented to participate in the long-term follow-up and a study visit occurred on 16 June 2023. Between 18 September 2019 and 16 June 2023, during the 45-month period since completing the primary study, she experienced 6 symptomatic UTIs, with positive urine cultures for gram-negative bacteria which included *E.coli, Klebsiella, Pseudomonas*, and *Acinetobacter.* No ESBL or carbapenem resistant species were reported. A patient interview was conducted during the study visit and participant 10-08-001 verbally communicated that she had experienced fewer UTIs and improved well-being after completing the vaccine series which was maintained during the long follow-up period. Periodically, during the long-term follow-up period, including during the study visit, she proactively inquired about the potential for booster vaccination.

### Participant 2: 10-08-002

At the time of screening for the Sequoia FimCH Vaccine EAS, participant 10-08-002, a 68-year-old female, presented with a history of multiple symptomatic urinary tract infections that began in March 2012. Her medical records documented 8 gram-negative culture-verified UTIs including multi-drug resistant ESBL-producing Enterobacteriaceae in the 12 months prior to enrollment to the study. These UTIs occurred despite receiving periodic antibiotic suppression therapy and she was therefore considered to be at risk for requiring intravenous carbapenem. Her medical history did not include any exclusionary comorbidities. No infections requiring antibiotics were reported in the 12-month pre-enrollment or during the pre and post vaccine other than UTIs.

Participant 10-08-002 was consented on 6 March 2018. A series of four FimCH vaccines were administered on 6 March 2018, 10 April 2018, 8 June 2018, and 30 August 2018 per protocol. During the 12-month study period from month 7 to month 19, 10-08-002 was diagnosed with 1 symptomatic UTI culture-confirmed for *Morganella.* Participant 10-08-002 received periodic UTI antibiotic prophylaxis during the post vaccine period. While the use of prophylactic antibiotic treatment cannot be excluded as contributory to reduction in UTIs, she received similar suppression therapy during the pre-vaccine period. Decrease in the number of symptomatic, culture-verified UTI occurrences post-vaccine correlates with observed peak anti-FimH antibody responses (**Figure 1**). She did not report any vaccine related adverse events, unexpected adverse events, or serious adverse events during study participation. She completed the EAS on 17 Sept 2019.

Participant 10-08-002 consented to participate in the long-term follow-up and a study visit occurred on 16 June 2023. Between 18 September 2019 and 16 June 2023, the 45-month period after completing the primary study, she experienced 5 symptomatic UTIs, with urine cultures for gram-negative bacteria including *E. coli*, *Pseudomonas*, *Morganella* and *Klebsiella* which were successfully treated with antibiotics including ciprofloxacin, cephalexin, and amoxicillin/clavulanate potassium. A patient interview was conducted the during study visit and participant 10-08-002 verbally reported she had experienced a decrease in the number of symptomatic UTIs in the 12 months after completing the vaccine series, followed by an increase between September 2020 and June 2023. She indicated that her general well-being improved post vaccine and this improvement was maintained for approximately 2 years. During the long-term follow-up period, including at the study visit, she proactively inquired about the potential for a booster vaccination.

### Participant 3: 10-08-003

At the time of screening for the Sequoia FimCH Vaccine EAS, participant 10-08-003, a 69-year-old female, presented with complaints of increasing frequency of symptomatic UTIs. In the 12 months prior to enrollment; she experienced 8 culture confirmed gram-negative multi-drug resistant UTIs; and was considered to be at risk for requiring intravenous carbapenem. Her medical history did not include any exclusionary comorbidities.

Participant 10-08-003 was consented on 6 March 2018. A series of 4 FimCH vaccines were administered on 6 March 2018, 17 April 2018, 5 June 2018 and 28 August 2018 per protocol. During the 12-month period from study month 7 to month 19, 10-08-003 was diagnosed with 1 symptomatic UTI, confirmed with a urine culture for *E.coli*, which was successfully treated with cephalexin. Additionally, she experienced a single episode of pneumonia in April of 2018 which required treatment with antibiotics. Decrease in the number of symptomatic, culture-verified UTI occurrences correlates with observed peak anti-FimH antibody responses (**Figure 1**). She did not report any vaccine related adverse events, unexpected adverse events or serious adverse events during study participation. She completed the initial EAS on 17 September 2019.

Participant 10-08-003 consented to participate in the long-term follow-up on 16 June 2023. During the 45-month period following completion of the initial study, she experienced 8 symptomatic UTIs, with positive culture results for *E.coli* and *Klebsiella*. She was successfully treated with antibiotic treatment. A patient interview was conducted during the long-term follow-up study visit and participant 10-08-003 stated that her general well-being improved post-vaccination. During the long-term follow-up period, including at the study visit, she proactively inquired about the potential for a booster vaccination.

### Participant 4: 10-09-001

At the time of screening for the Sequoia FimCH Vaccine EAS, participant 10-09-001, a 76-year-old male, presented with complaints of frequent symptomatic UTIs. His medical records documented 4 culture-verified UTIs in the 12 months prior to enrollment which included ESBL-producing *E. coli*. resistant to ceftriaxone, fluoroquinolones, and trimethoprim. One UTI progressed to pyelonephritis, bacteremia, and septic shock which required hospitalization and intravenous antibiotics. His past medical history was benign for exclusionary comorbidities. Other than UTIs, no infections requiring antibiotics were reported in the 12 months prior to study enrollment or in the pre or post vaccine period.

Participant 10-09-001 was consented on 30 October 2018. A series of 4 FimCH vaccines were administered on 30 October 2018, 4 December 2018, 29 January 2019, and 23 April 2019 per protocol. During the 12-month follow-up, from month 7 to month 19, he was diagnosed with 1 symptomatic UTI, confirmed by urine culture for *E. coli* which was successfully treated with antibiotic treatment. The decrease in symptomatic culture-verified UTI occurrences correlates with observed peak anti-FimH antibody responses (**Figure 1**). He did not experience any vaccine related adverse events, unexpected adverse events or serious adverse events during study participation. He completed the EAS on 23 April 2020.

Participant 10-09-001 consented to participate in the optional long-term follow-up on 25 July 2023. He reported no UTI occurrences during the long-term follow-up period. A patient interview was conducted during the study visit and participant 10-09-001 reported improvement in his overall quality of life which he attributes to the FimCH Vaccine. During the long-term follow-up period, including during the study visit, he proactively inquired about the potential for a booster vaccination.

## Immunogenicity

Anti-FimH antibody responses specific to the N-terminal region of FimH for the 4 participants included in this case report series were assessed from sera obtained pre-vaccination (day 0), at 1 month, 6 months, 7 months, 12 months and approximately 60 months post initial vaccine administration for each participant. Samples were analyzed using a qualitative research ELISA for the determination of anti-FimH IgG in serially diluted human serum samples and colorimetric reaction measured by optical density (OD450) using a VersaMax PLUS microplate reader and standard curve data analyzed by SoftMax Pro software. Average OD values were then normalized to each individual participant, not across participants, using Microsoft Excel.

Results of immunogenicity testing are summarized in **Figure 1** for normalized average OD for serum samples diluted 1:25,000. For all participants, data trends indicate that anti-FimH IgG titers progressively increased after each vaccine injection with peak antibody responses observed at month 7, approximately 1 month following the fourth vaccine in the series. By study month 12, approximately 6 months following the final vaccine in the series, anti-FimH IgG levels were declining and by the long-term follow-up visit of 5 years, the anti-FimH antibody titers were similar to pre-immunization levels.

## Statistical analysis

Due to the small number of participants in this case series, results are not generalizable to the full population. No formal hypotheses were planned or tested as part of the expanded access protocol. Study results are based on descriptive statistical analysis.

## Results

Four participants, 3 female and 1 male, aged 68-73, with a history of recurrent UTI considered at risk for requiring parenteral carbapenem treatment were enrolled in this expanded access FimCH Vaccine study. All four participants received four vaccine doses as scheduled per protocol. Safety and tolerability of the vaccine was consistent with what was reported in the Phase 1 population with no new site injection reactions or systemic adverse events noted (11). The total number of gram-negative culture-confirmed UTIs experienced by the 4 study participants was reduced by approximately 75% from the 12-month pre-study period as compared to the 12-month period following observed peak anti-FimH antibody response. Individual participant UTI reductions ranged from 40-88%. The reduction in symptomatic, gram-negative culture confirmed UTIs was maintained during the long follow-up period which ranged from 50-58 months. UTI occurrences during the 12-month pre-study enrollment period, the initial 12-month follow-up period and the long-term follow-up period are summarized in **Table 1**.

**Table 1:** Gram-negative culture confirmed UTI occurrences by participant and total population in 12 month period immediate prior to study enrollment, in 23-month period post FimH antibody peak response and during the long-term follow-up period.

UTI Occurrences and UTI Reduction Pre to Post Peak Ab Response	Participant 10-08-001	Participant 10-08-002	Participant 10-08-003	Participant 10-09-001	Total Population
Number of UTI occurrences pre study enrollment (12 month period)	5	8	8	4	25
Number of UTI occurrences post FimH peak Ab response Study Month 7-19 (12 month period)	3	1	1	1	6
Percent UTI Reduction	40%	88%	88%	75%	75%
Number of UTI Occurrences in 5-year long-term Follow-up Period ( 50-58 months of follow-up)	6	5	8	0	19

## Discussion

This expanded access study demonstrated the safety, immunogenicity, and reduction in UTI occurrences following immunization with the adjuvanted FimCH vaccine in 4 patients with recurrent UTI who were considered to be at risk for requiring intravenous carbapenem. The results are consistent with data generated in a Phase 1 population of participants previously reported. All 4 participants in this expanded access study were noted to have fewer symptomatic, gram-negative culture-verified UTIs in the 12- month period following administration of the fourth vaccine, and this was correlated with observed anti-FimH antibody response. Reduction in the annual total number of gram-negative culture positive UTI continued in the long-term follow-up period with UTI occurrences but began increasing approximately 2 years after completion of the vaccine series. Over time the anti-FimCH antibody titers trend toward pre-treatment levels suggesting vaccine boosters will be required to maintain durability of response. Vaccine booster requirements will be formally evaluated as part of all future Sequoia Vaccine FimCH Vaccine trials.

## Conclusion

This case report, conducted under a compassionate use IND, describes the safety, tolerability, immunogenicity, and occurrences of UTI in 4 patients at risk for requiring carbapenem for treatment of recurrent UTI. While the results cannot be extrapolated to the general population, this case report series supports that continued evaluation of the FimCH Vaccine is warranted and a Phase 2 protocol in up to 120 patients is planned.

## Data availability statement

The original contributions presented in the study are included in the article/**Supplementary Material**. Further inquiries can be directed to the corresponding author.

## Ethics statement

The studies involving humans were approved by Advarra IRB who is accredited by the Association for the Accreditation of Human Research Protection Programs (AAHRPP). The studies were conducted in accordance with the local legislation and institutional requirements. The participants provided their written informed consent to participate in this study. Written informed consent was obtained from the individual(s) for the publication of any potentially identifiable images or data included in this article.

## Author contributions

EP: Investigation, Writing – review & editing. HS: Investigation, Writing – review & editing. TE: Data curation, Project administration, Writing – review & editing. HH: Conceptualization, Data curation, Writing – review & editing. JL: Data curation, Formal analysis, Methodology, Validation, Writing – review & editing. EC: Data curation, Writing – review & editing. MO’N: Writing – review & editing. KB: Writing – review & editing. AK: Data curation, Formal analysis, Writing – review & editing. SH: Conceptualization, Writing – review & editing. TH: Writing – review & editing. JH: Conceptualization, Data curation, Formal Analysis, Investigation, Writing – review & editing.
